# Screening of BRCA1 (c.5177_5180delGAAA rs80357867 and c.4986+6T>C rs80358086) and the BRCA2 (c.6445_6446delAT rs80359592) Genes for Breast Cancer Prevention in Burkina Faso

**DOI:** 10.4314/ejhs.v32i4.5

**Published:** 2022-07

**Authors:** Isabelle T Kiendrebeogo, Abdou A Zoure, Fabienne I Zongo, Serge Y Ouedraogo, Alexis Y Sawadogo, Jospin Amegnona, Herman K Sombie, Jean T Valérie Elvira Bazie, Pegdwendé A Sorgho, Albert T Yonli, Marie N Lamoussa Ouedraogo, Dorcas Obiri-Yeboah, Nayi Zongo, Hierrhum A Bambara, Jacques Simpore

**Affiliations:** 1 Laboratory of Molecular Biology and Genetics, UFR/SVT, University Joseph KI-ZERBO, Burkina Faso; 2 Pietro Annigoni Biomolecular Research Center, Burkina Faso; 3 Institute of Health Sciences Research, Department of Biomedical and Public Health, Burkina Faso; 4 Department of General and Digestive Surgery, University Hospital Center Yalgado Ouedraogo, University Joseph KI-ZERBO, UFR/SDS, Burkina Faso; 5 Service of oncology, University Hospital Center Bogodogo, University Joseph KI-ZERBO, UFR/SDS, Burkina Faso; 6 Service of Gynecology, University Hospital Center Bogodogo, University Joseph KI-ZERBO, UFR/SDS, Burkina Faso; 7 Faculty of Health Sciences, University Saint Thomas d'Aquin; 8 Department of Microbiology and Immunology, School of Medical Sciences, University of Cape Coast

**Keywords:** rs80357867, rs80358086, rs80359592, BRCA, genes, Burkina Faso

## Abstract

**Background:**

The objective of this study is to search for mutations in the BRCA1 (c.5177_5180delGAAA and c.4986+6T>C) and BRCA2 genes (c.6445_6446delAT) in a population of women diagnosed with breast cancer.

**Methods:**

This is a case-control study that involved 140 participants, including 70 patients with histologically diagnosed breast cancer and 70 healthy women without breast cancer. Mutations in the BRCA1 (rs80357867, rs80358086) and BRCA2 (rs80359592) genes were tested by real-time PCR. The 95% confidence interval Odds Ratio (OR) was used to estimate the associations between specific genotypes and breast cancer.

**Results:**

The study revealed that no mutations were detected for rs80359592. Similarly, no reference allele (TTTC/TTTC) of rs80357867 was found in this study. However, the homozygous double mutant (-/) genotype of this rs80357867 was observed in 11.43% and 1.43% of patients and controls respectively, while 88.57% of patients and 98.57% of controls had a heterozygous deletion (TTTC/-). Concerning rs80358086, 8.57% of the patients had a heterozygous mutation (A/G) with no significantly risk association with occurrence of breast cancer (OR = 6.46; 95% CI: 0.75–55.21; p = 0.11). In addition, this heterozygous mutation was significantly associated with a family history of breast cancer (OR=128; 95% CI: 9.46–1730.93) and breast cancer risk in nonmultiparous women (OR=6; 95% CI: 1–35.90; p= 0.05) but no association with overweight/obesity (OR=1.66; 95% CI: 0.18–15.35; p=1).

**Conclusion:**

This study shows high frequencies of heterozygous mutation of rs80357867 and rs80358086 from patients. In Burkina Faso, these results could help with early diagnosis of breast cancer in patients.

## Introduction

Cancer is a global public health problem. In 2020, the world recorded 19.3 million new cases with 10 million deaths. Female breast cancer is the most frequently diagnosed cancer with 2.3 million (11.7%) new cases and 684,996 deaths (6.9%) in 2020. It is also the fourth most deadly cancer (1). Africa and Polynesia have the highest breast cancer mortality rates. According to WHO 2020, in sub-Saharan Africa (SSA), half of the women who died from breast cancer were under 50 years old. In Burkina Faso in 2020, it reached 1927 new cases (16%) and 1142 deaths (13.1%) making it the most diagnosed and the second deadly cancer (2). In women, it is the leading cancer in terms of both incidence and mortality (2).

Breast cancer is a multifactorial and complex disease. Approximately 5–10% of breast cancer cases are due to a genetic and hereditary problem caused by mutations in high penetrance susceptibility genes. Many other factors, including environmental, hormonal and viral factors appear to be important in the mechanism of these risk factors (3).

Radiation exposure, gynecological history (age at menarche, first pregnancy, etc.), smoking, and postmenopausal hormone therapy appear to be involved in breast cancer development. A family history of breast cancer increases the risk of breast cancer (4). The majority of women diagnosed with breast cancer have no known family history. However, the World Health Organization (WHO) emphasizes that the absence of a known family history does not necessarily mean that a woman has a lower risk of developing breast cancer.

Inherited “high penetrance” genetic mutations greatly increase the risk of breast cancer. Approximately 5% to 21.5% of breast cancers arise from germline mutations associated with genes such as *BRCA1, BRCA2, p53* and *PTEN*, which puts an individual at risk of developing hereditary breast cancer (5). Patients with positive expression of the *BRCA1* and *BRCA2* genes have about an 80% risk of developing breast cancer later in life, mainly around pre-menopausal age (6). The prevalence and spectrum of germline mutations in the *BRCA1* and *BRCA2* genes are certainly better delineated in European and North American population (7).

For many of these populations, recurrent and founder mutations have been identified and this has allowed the development of targeted genetic tests which, being less expensive, can be more easily used for population screening (8).

For example, the mutation c.5177_5180delGAAA (p.Arg1726Lysfs*3) rs80357867 (c.5177_5180delGAAA, exon18) is located in exon 18 of the *BRCA1* gene and is also known as 5296del4. It is a 4 base pair deletion of exon 18 of BRCA1 mRNA and is considered pathogenic for breast cancer (9). It causes a reading frame shift that alters the amino acid sequence of the protein starting at position 1726 and leads to a premature termination codon 3 amino acids downstream. This alteration leads to a non-functional truncated protein (9). Heterozygous loss of function of the *BRCA1* gene is an established pathological mechanism in hereditary breast and ovarian cancer (HBOC) (10). This variant has been reported in many people with breast and/or ovarian cancer (11) and was also identified in 2/251,090 chromosomes in the general population by the genome aggregation database (gnomAD). In addition, it has been identified in 1/10,314 African chromosomes by the Exome Aggregation Consortium (ExAC, http://exac.broadinstitute.org; dbSNP rs80357867).

Furthermore, the intronic mutation (c.4986+6T>C, exon15) rs80358086 in the *BRCA1* gene has a significant impact on splicing as it leads to activation of a cryptic downstream splice donor site, resulting in an aberrant RNA transcript and a truncated protein. It is found in patients with hereditary breast and ovarian cancer (12). Known as IVS16+6T>C, this sequence change occurs 6 nucleotides after exon 15 of the BRCA1 gene. This position is conserved in the human genome and has a crucial role in mRNA processing. This mutation results in incorrect splicing, altered reading frame and truncated protein creating breast cancer (13).

Finally, the c.6445_6446delAT (p.Ser248Ile2149*) rs80359592 (c.6445_6446delAT, exon11) mutation located in exon 11 of the *BRCA2* gene is a frame shift mutation. It results in the replacement of serine by Isoleucine and a translation stop leading to a nonfunctional truncated protein. Loss-of-function variants of *BRCA2* are known to be pathogenic (10). This variant has been reported in people with breast cancer (14).

However, lack of resources for preventive screening and access to quality care result in significant delays in breast cancer detection, contributing to a high mortality rate in West Africa (15, 16).

Furthermore, the genetics of breast cancer in African countries are generally unknown (19) and although the early age of onset and aggressiveness suggest that there may be a strong hereditary component in the development of the disease, few studies have attempted to make up for this lack of knowledge (17–18).

Data are scarce for most West African countries and the results of the few studies carried out do not provide a true distribution of country-specific mutations.

The aim of this study was therefore, to determine the prevalence of (rs80357867, rs80358086) of the *BRCA1* gene and (rs80359592) of the *BRCA2* gene, with a view to contributing to breast cancer prevention in Burkina Faso.

## Materials and Methods

**Study population**: This was a matched case-control study, conducted between December 2020 and June 2021, at the University Hospital Centres: Yalgado Ouédraogo (CHU-YO) and Bogodogo (CHU-B) and at the medical centres (Paul VI and Schiphra). It enrolled 70 patients histologically diagnosed with breast cancer confirmed by a pathologist and 70 controls without breast cancer, who came for medical consultation due to reasons other than breast cancer, had no family history of breast cancer, had mammograms and were declared healthy with respect to breast cancer.

**Sample collection**: After obtaining free and informed consent from patients and controls, a questionnaire was administered to collect sociodemographic, anthropometric and clinical data from the participants. Venous blood was collected in two 2 mL EDTA (Ethylene-Diamine-Tetra-Acetic) tubes from the participants. After centrifugation at 1500 rpm for 15 minutes, the plasma and the pellet were separated and stored at -20°C at the Pietro Annigoni Biomolecular Research Center (CERBA).

**Characterization of mutations by RT-PCR**: Genomic DNA was extracted from the pellet using the Qiagen kit, product of Germany (QIAamp® DNA Mini Kit (50) cat.No.51304) and followed the manufacturer's protocol. The purity and concentration of the resulting DNA were determined using Biodrop µLITE (Isogen Life Science, Temse, Belgium). The extracted DNA was stored at -20° C.

Mutation genotyping was performed by RT-PCR method with the Applied Biosystems™ QuantStudio ™5 instrument. The total reaction volume was 20µL which contains 8µL of nuclease-free water, 7 µL of the Master Mix (Applied Biosystems™ Taqman™ 2X Universal PCR Master Mix, No AmpErase™ UNG, REF 4324018), 1 µL TaqMan® SNP genotyping primers (10X) (**Table 1**), and 4 µL of DNA (80 µg/ml).

**Table 1:** Specific primer pairs for real-time PCR amplification (Catalogue number: 4351379)

Gene	NM	coding	protein	dbSNP	Primer :5′-3′ (F : Forward, R:Reverse	Annealing temperature
BRC A1	NM_007 294.3	c.5177_5180d elGAAA	p.Arg1726 Lysfs*3	_153129647_2 0 rs80357867	BRCA1ex18 F :TTAAAGGGCTGTGGCTTTAG BRCA1ex18 R:AAGGAAAGTGGTGCATTGAT	57 °C
BRC A1	NM_007 294.3	c.4986+6T>C	/	rs80358086 C_153129717_ 10 rs80358086	BRCA1ex15 F:AACAGAGACCAGAACTTTG BRCA1ex15 R:AAACTCTTTCCAGAATGTTG	54 °C
BRC A2	NM_000 059.3	c.6445_6446d elAT	p.Ile2149*	C_154785231_ 20 rs80359592	BRCA2ex11 F:AAACCCAGAGCACTGTGTAAACTC BRCA2ex11 R:TCTCCTCTTCTTTTTCCAATTCTTG	61 °C

Genotyping was done by TaqMan allelic discrimination tests with probes labelled with FAM/VIC fluorophores. The different alleles were associated with the fluorochromes VIC for the reference allele and FAM for the alternative allele. The amplification programme consisted of an activation phase at 95°C for 10 minutes, followed by 50 cycles of denaturation series at 94°C for 30 seconds, hybridisation at 60°C for 60 seconds, and elongation at 60°C for 60 seconds.

**Statistical analysis**: Study data were entered into Excel 2013 and processed with Statistical Package for the Social Sciences (SPSS) version 21. Results were considered statistically significant at p ≤ 0.05, using Fisher's exact test. The Odd Ratio (OR) and 95% confidence intervals (CI) were calculated to estimate associations between cases and controls using Epi Info version 7.1. Allelic discrimination was performed using QuantStudio™ Design & Analysis Software v1.5.

**Ethical Considerations**: Our study obtained approval from the Health Research Ethics Committee (CERS) of Burkina Faso (Deliberation No. 2019-5-067 of 15 May 2019). All participants gave their free and informed consent. Confidentiality and anonymity regarding the information provided were respected. An information and consent form was given to each participant before the collection.

## Results

**Sociodemographic and clinical characteristics of the study population:** Table 2 shows the sociodemographic and clinical characteristics of the population. The mean age of cases and controls was 48 ±11.53 years [23 to 73 years] and 36.98 ±1.24 years [21 to 65 years], respectively. The majority (44.28%) of cases were less than 45 years' old (Table 2).

**Table 2:** Sociodemographic and clinical characteristics of the study population

Characteristic	Controls n = 70 (50%)	Cases n = 70 (50%)
**Age (years)**		
<30	22(31.43%)	5(7.14%)
30–45	33(47.14%)	26(37.14%)
46–60	12(17.14%)	29(41.43%)
>60	3(4.29%)	10(14.29%)
**BMI (kg/m^2^)**		
< 18,5 (Underweight)	2(2.86%)	2(2.86%)
18,5 < IMC ≤ 24.9 (Normal)	30(42.86%)	15(21.43%)
25 ≤ IMC ≤ 29.9 (Overweight)	20(28.57%)	29(41.43%)
≥30 (Obeses)	18(25.71%)	24 (34.28%)
**Number of children**		
Multiparous	37(52.86%)	50(71.43%)
Non multiparous	33(47.14%)	20(28.57%)
**Residence**		
Rural	3(4.29%)	11(15.71%)
Urban	67(95.71%)	59(84.29%)

Body mass index (BMI) was calculated as the ratio of weight to height squared. These indices were then grouped into underweight <18.5 kg/m2; Normal (18.5 < BMI ≤ 25 kg/m2); Overweight (25 and 29.9 kg/m2); and Obese (≥ 30 kg/m2) according to US National Institute of Health/National Heart Lung and Blood Institute (NCI/NHLBI) criteria. Women with four (4) to six (6) deliveries were considered as simple multiparous, while a grand-multiparous had at least seven (7) and more childbirth. These two groups were designated as multiparous. Nulliparous (no delivery), primiparous (one childbirth) and pauciparous (two to three deliveries) were designated as non-multiparous.

Table 3 shows that the prevalence of overweight and obesity was 28.57% and 25.71% in controls and 41.43% and 34.28 in cases, respectively. However, 32.14% of the participants had a normal Body Mass Index (BMI).

**Table 3:** Genetic characteristics of the study population

Characteristics	Contols n = 70	Cases n = 70	OR (95%) CI	*p*-value
**rs80357867**				
TTTC/TTTC (Homozygous reference allele)	0(0%)	0(0%)	NA	NA
TTTC/- (Heterozygous mutated	69(98.57%)	62(88.57%)	NA	NA
-/- (Homozygous alternative allele)	1(1.43%)	8(11.43%)	NA	NA
**rs80358086**				
A/A (Homozygous reference allele)	69(98.57%)	64(91.43%)	Ref	-
A/G (Heterozygous alternative allele)	1(1.43%)	6(8.57%)	6.46 (0.75–55.21)	0.11
G/G (Homozygous alternative allele)	0(0%)	0(%)	NA	NA
HWE	1	0.89		
**rs80359592**				
TA/TA (Homozygous reference allele)	70(100%)	70(100%)	Ref	-
TA/- (Heterozygous alternative allele)	0(0%)	0(0%)	NA	**NA**
-/- (Homozygous alternative allele)	0(0%)	0(0%)	NA	**NA**

**OR**: Odds ratio; **95%: CI** Confidence Intervalle at 95%; ***p***: *p*-value; **Ref**: Reference; **NA**: Not applicable; HWE: Hardy-Weinberg Equilibrum

**Figure 1:**
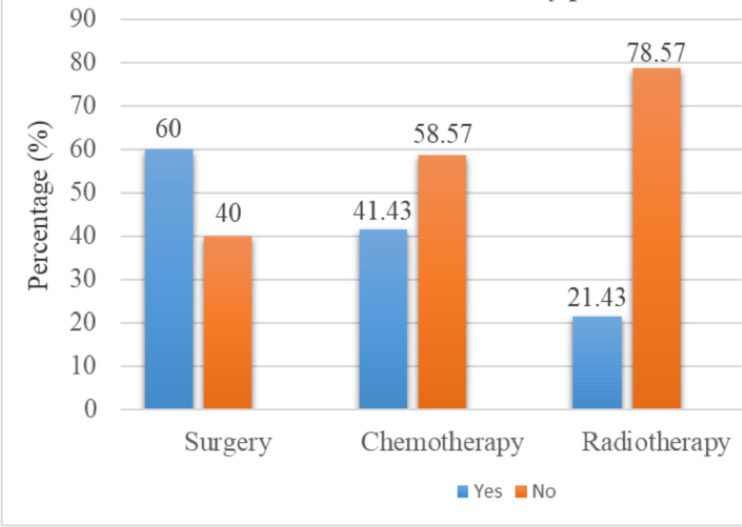
Percentage of patients by treatment received.

**Treatments received by patients**: Among the cases, 29 or 41.43% received chemotherapy, 15 or 21.43% received radiotherapy, 15 or 21.43% received both treatments (chemotherapy and radiotherapy) and 27 or 38.57% received no treatment. Also breast surgery was performed in 42 patients (60%) (Figure 1).

**Family history of breast and other cancers**: In this study 14.28% of cases reported a family history of cancer of which 5.72% represented familial breast cancer and 7.14% other types of cancer (cervical, bladder, vulva, colon, liver, prostate).

**Allelic carriage of** rs80357867 **(c.5177_5180delGAAA), rs80358086 (c.4986+6T>C) of the *BRCA1* gene and rs80359592 (c.6445_6446delAT) of *BRCA2* gene**: In our study, no mutations were found for the *BRCA2* gene rs80359592. For the *BRCA1* gene (rs80357867), the results show that 62 patients (88.57%) had a heterozygous mutation and 8 (11.43%) a homozygous mutation; while 69 controls (98.57%) had a heterozygous mutation and one (1.43%) a homozygous mutation.

For the *BRCA1* gene (rs80358086) we had 6 (8.57%) heterozygous mutations and homozygous mutations in patients; in controls, one heterozygous mutation (1.43%) was detected (Table 3). The heterozygous mutation in rs80358086 was associated with a non-significant risk of breast cancer occurrence (OR = 6.46; 95% CI: 0.75–55.21; *p* = 0.11). We were only able to check the Hardy-Weinberg equilibrium for rs80358086. Indeed, there were no carriers of the reference allele genotype of rs80357867. In addition, no mutations were observed for rs80359592.

**Association between clinical parameters and genotypic expression of rs80358086**: Given that reference allele mutation of rs80359592 and reference genotype of rs80357867 were not observed in this study, this association of variables was made only with rs80358086. Heterozygous mutation of rs80358086 was significantly associated with a family history of breast cancer (OR=128; 95% CI: 9.46–1730.93; *p*<0.001. Regarding BMI, the presence of the heterozygous mutation in rs80358086 was not associated with overweight/obesity (OR=1.66; 95% CI: 0.18–15) 35; *p*=1. The presence of the heterozygous mutation in rs80358086 was significantly associated with the development of breast cancerin non-multiparous women with respectively (OR=6; 95% CI: 1–35.90; *p*= 0.05) (Table 4).

**Table 4:** Association between family history, parity, obesity, and genotypic expression of rs80358086 in cases

rs80358086	Family history of breast cancer		OR (95%) CI	*p*-value
			
	Yes (5)	No (65)		
A/A	1	64	Ref	-
A/G	4	2	128(9.46–1730.93	< **0.001**
G/G	0	0	NA	**NA**
**rs80358086**	**Parity** **No multiparous** **(20)**	**Multiparous(50)**		
A/A	16	48	Ref	-
A/G	4	2	6(1–35.90)	**0.05**
G/G	0	0	NA	**NA**
**rs80358086**	**Obesity** **Normal (17)**	**Overweight/Obese (53)**		
A/A	16	48	Ref	-
A/G	1	5	1.66 (0.18–15.35)	**1**
G/G	0	0	NA	**NA**

**OR**: Odds ratio; **95%CI**: Confidence Intervalle at 95%; ***p***: *p*-value; **Ref**: Reference; **NA**: Not applicable

## Discussion

**Sociodemographic data**: Our study population is marked by its youth with 59% having an age ≤ 45 years. The average age of the cases was 48 ±11.53 years and is comparable to that found in 2019 in Burkina Faso, i.e. 48.20 ± 12.4 years (19). The mean age years is also similar to those reported in Mali: 47.7 ± 12 years (20), and in general among Arab populations (48 years) (21). However, this average is low compared to other countries such as France, the United States and Canada, where breast cancer is a disease of older women (about 63 years old and only 25–30% of patients are under 50) (22). This can be linked to the youthful age of the Burkinabe population as a whole. Indeed, 77.9% of the Burkinabe population is under 35 years old (23). Since 1997, the 41–50 age group has been identified as the most affected by breast cancer in Burkina Faso (24). We found 65% of overweight/obese women in our study. While some studies have associated obesity with an increased risk of breast cancer (25), a study on breast cancer risk factors in Burkina Faso found that 18.75% of their patients were overweight and that being overweight increased the risk of developing cancer for non-multiparous women (26).

Multiparity in our study was found in 71.43% of patients. A study conducted in Burkina Faso in 2016 had shown that this protective effect seems to increase proportionally to the number of deliveries (26). In our study, 90% of participants lived in urban areas, including 84.9% of patients. Our data are comparable to those of 87.50% found in studies in 2019 in Burkina (19). This could be explained by the fact that the study recruitment was done in town (Ouagadougou) and also by the fact that women in rural areas have difficulty consulting a health practitioner for their health problems (27).

**Family history of breast and other cancers**: In this study 14.28% of cases had at least one family history of cancer, among them 5.72 represented breast cancer, 1.43% represented familial breast cancer plus other types of cancer (cervical, bladder, vulva, colon, liver, prostate); 7.14% other types of cancer. Our data is lower than the 18.75% found in 2016 and 2019 in Burkina Faso (19, 26).

Family history is associated with an increased risk of breast cancer (18). The risk is higher in younger women and when the disease has developed in a close relative (mother, daughter or sister), before the age of 50 (28). In Western countries, approximately 13% of breast cancer patients have a family history related to (29). In addition, certain genetic mutations may increase the risk of breast cancer. Two genes, *BRCA1* and *BRCA2*, seem to be the most involved (30). Compared to the general population, women with mutations in these genes have an increased risk of breast cancer (28).

**Genetic characteristics of the study population**: *BRCA1* or *BRCA2* mutations may be responsible for approximately 10% of ovarian cancer cases and 3–5% of breast cancer cases. The estimated carrier frequency is 1 in 300 for *BRCA1* and 1 in 800 for *BRCA2* in the general population, with the exception of Ashkenazi Jewish women, who have a carrier frequency of 2% to 5% of founder mutations in *BRCA1* and *BRCA2* (31). In our study we did not obtain mutations for the *BRCA2* gene, rs80359592 (c.6445_6446delAT, exon11). For the *BRCA1* gene, rs80357867 (c.5177_5180delGAAA, exon18) the study revealed 69 (98.57%) heterozygous mutations and one (1.43%) homozygous mutation in the controls; and 62 (88.57%) heterozygous mutations and 8 (11.43%) homozygous mutations in the cases for the *BRCA1* gene, rs80358086 (c.4986+6T>C, exon15) in cases 6 (8.57%) homozygous mutations were found and in controls, one heterozygous mutation was detected.

The mutation rs80357867 c.5177_5180delGAAA (p.Arg1726Lysfs*3) is considered pathogenic for breast cancer (ClinVar), as it codes for a truncated non-functional protein. This variant has been reported in individuals and families with breast and/or ovarian cancer (9, 32). The p.Arg1726fs variant of *BRCA1* has been found in more than 30 individuals with *BRCA1*-related cancers (33).

In our study, there were no carriers of the reference genotype of this variant. However, high frequencies of the heterozygous mutation of this variant were found. The strong presence of this variant in the population puts women at risk of breast cancer as this variant is known to be pathogenic.

The pathogenic intronic mutation rs80358086 c.4986+6T>C results from a T to C substitution 6 nucleotides after coding exon 15 in the *BRCA1* gene. This alteration has been identified in several patients with early breast cancer (34) and also in ovarian cancer cohorts (12). Analysis of RNA from a breast/ovarian cancer family showed that this mutation activates a cryptic splice donor site, resulting in insertion, frameshift and premature truncation (13). Experimental studies have shown that this sequence change creates an aberrant transcript by activating a cryptic splice donor site. The transcript incorporates 65 bases of intronic sequence immediately after exon 15, resulting in a stop codon at residue 1676 (p.Met1663Valfs*14) (13). In our population, this variant was associated with a non-significant risk of breast cancer occurrence. This could mean that this mutation requires other factors to trigger the disease.

The rs80359592 c.6445_6446delAT (p.Ser248Ile2149*) mutation in the *BRCA2* gene results in a sequence change that creates a premature translation stop signal (p.Ile2149*). This results in a truncated protein product. This variant has been found in people with breast cancer or suspected breast/ovarian cancer (35). Mutations in the tumor suppressor gene *BRCA2* are associated with a predisposition to breast and ovarian cancers. *BRCA2* has a central role in maintaining genome integrity by facilitating the repair of toxic DNA double strand breaks (DSBs) through homologous recombination (HR) (36).

The absence of this mutation does not mean that *BRCA2* is not associated with breast cancer in Burkina Faso. There is a need to investigate other mutations in this gene in a much larger population. In Burkina Faso, prior to this study, previous studies have addressed BRCA. A study conducted in Burkina Faso in 2017 on the c.68_69delAG (exon2), c.181T>G (exon5), c.798_799delTT and 943ins10 (exon11) mutations of the BRCA1 gene found no carriers (18). In addition, the following BRCA1 mutations: A/G (c.4837A>G rs1799966; c.3548A>G rs16942; c.3113A>G rs16941; c.3418A>G rs2227945), C/T (c.2612 C>T rs799917); and BRCA2: A/G (c.7319A>G rs4986860), C/T (c.7397C>T rs169547) were identified in a study in Burkina Faso but all these variants are missense mutations with benign clinical significance (17).

**Association between family history, parity, obesity, and the rs80358086 mutation**: To date, little data is available on genetic risk factors in African populations. We also did not find any articles in the literature that address clinical parameters with this rs808806 mutation. We found in our study that the rs80358086 mutation was significantly associated with a family history of breast cancer and breast cancer risk in nonmultiparous. Family history is consistently associated with an increased risk of breast cancer (22). The family history of breast cancer has not been well studied in African studies, although several studies have shown it to be an important etiological factor. A family history of breast cancer is associated with a risk of developing breast cancer in proportion to the degree of kinship. Indeed, oncogenic genes, such as *BRCA1* and *BRCA2*, are responsible for most hereditary cases, and are associated with a high risk, whereas mutations in *TP53* are responsible for most sporadic cases (but can also be mutated in the familial case: Li-Fraumeni syndrome).

Multiparity is a factor that is associated with protection against breast cancer. Indeed, women who have had seven or more births have a 30% lower risk than those who have had fewer births (6, 28). Unfortunately, this protective effect is null after the menopause, leading to an increased risk of breast cancer (26).

In previous studies, African women are characterised by an advanced age at menarche (after 15 years); primiparity at a relatively early age (around 19 years) and by multiparity (5 to 9 children per woman) which would protect them from breast cancer (37). However, the adoption of Western lifestyles is now contributing to a decline in these protective factors for African women (38). In addition, the average age of primiparity was very close to 24.04 years and the average number of children per woman was 2.39 children. These trends of late primiparity and low parity could partly explain the increase in breast cancer incidence in Burkina Faso (39).

The rs80358086 mutation was associated with overweight/obesity with a non-significant risk. Obesity is a factor that increases the risk of breast cancer (25). Generally speaking, it is known that African women are plump, so much so that several study cohorts, notably in Nigeria, have not found a risk of breast cancer linked to obesity (40).

The prevalence and spectrum of germline BRCA mutations are not yet well defined in West Africa. Data are scarce for most countries and the results of the few studies that have been done do not give a true picture of country-specific mutations. However, in European and North American populations this is well-established (7).

These countries have a mapping of recurrent and founder mutations allowing the development of targeted genetic tests which, being less expensive, can be more easily used for population screening (8). It is therefore important for Africa to identify and characterize recurrent mutations, and develop mutation panels for specifics populations (18).

One of the limitations of our study is the lack of data on certain socio-demographic and clinical variables (histological type, grade and stage of their cancer) of patients. These points to the need for a cancer registry in Burkina Faso. The other is the small size of our study population.

Our study shows high frequencies of the heterozygous deletion of rs80357867 and the heterozygous mutation of rs80358086 in the patients. Furthermore, the heterozygous mutation of rs8038086 was associated with a family history of breast cancer and non-multiparty, which is a risk factor for breast cancer.

In the context of resource-limited countries, these analyses allow for early detection of pathogenic mutations and for rigorous follow-up and better management. Our results could be used to help in the early diagnosis of breast cancer in Burkina Faso, both in patients and in their first, second and third degree relatives (daughter, sister, aunt).
